# An Ultra-Wide Load Range Voltage Converter Using Proactive Phase Frequency Modulation for IoT Sensors

**DOI:** 10.3390/s20216279

**Published:** 2020-11-04

**Authors:** Saad Arslan, Syed Asmat Ali Shah, HyungWon Kim

**Affiliations:** 1 Department of Electronics Engineering, Chungbuk National University, Chungdae-ro 1, Seowon-gu, Cheongju 28644, Korea; saad@chungbuk.ac.kr; 2 Department of Electrical and Computer Engineering, COMSATS University Islamabad, Park Road, Tarlai Kalan, Islamabad 45550, Pakistan; 3 Department of Electrical and Computer Engineering, COMSATS University Islamabad, Abbottabad Campus, University Road, Tobe Camp, Abbottabad 22060, Pakistan; asmat_ali@cuiatd.edu.pk

**Keywords:** switched capacitor, voltage converter, wide load range, multiphase operation, variable frequency

## Abstract

Modern sensor nodes have multiple operating states, which causes a conventional voltage converter to perform poorly over a wide load range of the operating states. This paper proposes a voltage converter whose switching frequency and output voltage are proactively adjusted to maintain high conversion efficiency. This allows the converter to exploit a wider frequency range to cover a wide load range. In addition, the proposed converter uses multiple smaller capacitor banks and employs multiphase operation to provide low output ripple voltage. A distributed topology for non-overlapping signal generation is proposed and used in the converter to minimize the number of wires running from connecting the controller to the converter. The proposed voltage converter has been implemented in a chip using a 0.13 um CMOS process. The measurement results demonstrate the ability to support a wide load range of 10 µA to 10 mA, for switching frequencies ranging from 100 kHz to 200 MHz, while providing an efficiency of above 80%.

## 1. Introduction

**Figure 1:**
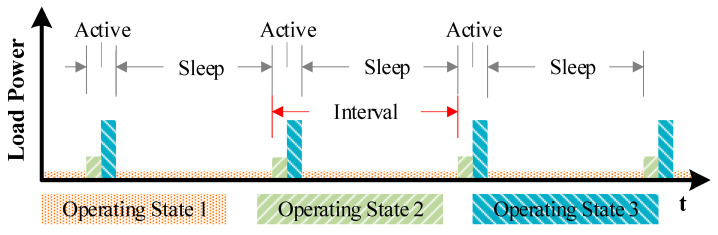
Load behavior with multiple operating states, repeated with a schedule/interval.

Modern sensor nodes perform a set of tasks repeatedly, and thus often exhibit periodic transition between different load states based on a schedule [1,2,3], as shown in Figure 1. The predictable transition schedule allows the software to proactively reconfigure the voltage converter to supply required amount of current for various load conditions. Under varying load conditions, however, a large Switched Capacitor (SC) converter often provides poor efficiency at smaller loads. It is known that, in recent mobile platforms, around 32% of the overall power is wasted, in step-down conversions [4]. This is because most voltage converters are optimized targeting the average load condition, and thus perform poorly under peak and minimum load conditions.

Another challenge faced by modern SC converters is the large output ripple voltage, which is often alleviated by having a large output capacitor and/or high switching frequency. These approaches to reducing ripple result in large area overhead and high switching losses, respectively. In [5], a dithered capacitance modulation scheme is presented to reduce ripple at the expense of much higher oscillator frequency than the converters’ switching frequency and circuit complexity. A simpler and effective approach to reduce ripple is splitting a converter into smaller units and operating the units at a fixed phase difference [5,6,7]. A multi-phase converter, due to its low ripple, can offer high conversion efficiency as well. For example, a multiphase operation equally distributes the current surges from the input capacitor, over time. This reduces the ripple voltage ΔV = V_Max_ − V_Min_ on the input capacitor. It is known that charging a capacitor with small voltage difference results in high energy efficiency in charge transfer process between the supply and capacitor voltage [8].

Under light load conditions, the smaller units can be individually turned-off to reduce losses [9]. However, turning off some of the capacitor banks operating at multiphases causes the output ripple voltage to increase unevenly. Therefore, to fully benefit from a converter using multiphase, all capacitor banks must be operational at all times. A popular technique to reduce switching losses is utilizing Pulse Frequency Modulation (PFM), which reduces the switching of the converter depending on the load [6,10,11]. Most of existing PFM methods are reactive (act in response to an event), which change the switching frequency based on the output voltage feedback. This results in a slower response and failure to utilize a wide frequency range, which degrades conversion efficiency at light loads. Furthermore, PFM methods (especially when combined with multi-phase) generate low frequencies from a high-speed oscillator with a fixed phase difference. Therefore, in PFM methods, the control circuit’s power consumption dominates for small load current leading to poor conversion efficiency [10].

Multi-phase voltage converters (comprising of multiple units) need a large number of switches and associated control signals. Therefore, it is infeasible to apply the conventional techniques for non-overlapping signal generation, since they adopt a centralized approach [9,11] and need a large number of wires. Due to the large number of wires, the conventional techniques incur disadvantages such as large area, high power consumption, switching uncertainty and susceptibility to noise. The researchers of [5] suggest reducing the number of phases to reduce the power consumption induced by non-overlapping signals and other switching circuits. A distributed non-overlapping scheme is presented in [6], where an additional NMOS or PMOS is added in the MOSFET driver circuit to delay the turning-on. However, adding an additional NMOS or PMOS in the last stage of the driver would add significant area overhead and slowly turn on the MOSFET (in additional to the delay).

This paper proposes a multi-phase SC converter targeting sensor nodes that operate in predictable schedules of alternating active and sleep periods exhibiting a wide range of load as shown in Figure 1. The converter maintains high conversion efficiency by proactively adjusting its switching frequency, using an integrated oscillator, based on the schedules of load current demanded by the target load. This approach allows the converter to operate with a wider frequency range than existing PFM designs. The proposed distributed non-overlapping signal generation is realized in the MOSFET drivers by delaying the turn-on transition with minimal area overhead and reduced power consumption.

Section 2 presents the proposed converter in more detail. Section 3 discusses the implementation of a test chip to evaluate the proposed converter. Section 4 describes the test setup and demonstrates the converter’s performance measurements. Section 5 discuss the measurement results and compares with other works. Finally, concluding remarks are presented in Section 6.

## 2. Proposed SC Voltage Converter

### 2.1. Architecture

**Figure 2:**
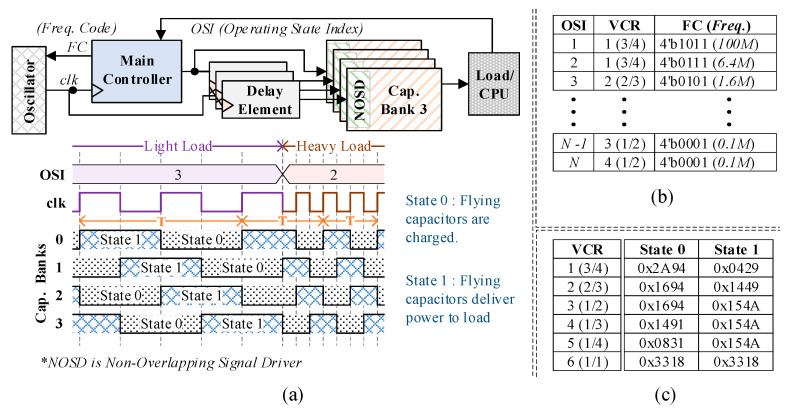
Proposed Switched Capacitor (SC) converter: (**a**) block diagram and operation; (**b**) an example Operating State Index (OSI) table in the controller; (**c**) Voltage Conversion Ratio (VCR) table specifying control signals for each state.

This paper proposes a voltage converter that offers high conversion efficiency over a wide range of loads and reduced ripple voltage. To deliver a wide load range, the operating frequency of the converter is adjusted by power management software based on the task scheduling prediction. To provide reduced ripple voltage, the SC voltage converter is split into multiple units (capacitor banks) that are operated with a predefined phase difference. Figure 2a illustrates the overall structure of the proposed voltage converter which consists of an oscillator, main controller, delay elements and multiple capacitor banks. Here, each capacitor bank includes a set of Non-Overlapping Signal Drivers (NOSDs), which can minimize the switching losses at very low cost of circuit overhead.

The SC converter in Figure 2a has four identical capacitor banks, which operate at a phase difference of T/4. Each capacitor bank toggles between two states: state0 (charging) and state1 (delivery). Thus, one period of converter operation comprises of two clock cycles; see Figure 2a. All the capacitor banks share one controller, while each delay element defers the control signal for individual capacitor bank by T/4.

### 2.2. Operation

Before the SC converter starts a normal operation, its main controller is pre-programmed with the sequence of the operating states of the load. Figure 2b shows an example of such a program for operation states. The program stores a sequence of Operating State Indexes (OSI), which contains the target Voltage Conversion Ratio (VCR) and Frequency Code (FC). The VCR indicates the ratio of the target output voltage to the input voltage. The FC in each entry in the OSI table is programmed to match the target load current for the corresponding operating state. To configure the proper voltage ratio, the main controller stores a VCR table, which is shown in Figure 2c. Each entry of the VCR table provides control signals for the two states of the capacitor banks, e.g., state0 (charging) and state1 (delivery). 

When the power management software in the load CPU of sensor nodes is about to change its operating state, it sends its operating state index (OSI) to the controller of the converter. The main controller looks up the OSI table and retrieves VCR and the FC for the corresponding OSI. The VCR is passed to the VCR table to retrieve the control signals for each state of the capacitor bank. The FC is provided to the oscillator to generate the frequency that allows the converter to supply the required current. The main controller maintains its current frequency and VCR, until a new OSI is received from the power management software.

### 2.3. Oscillator

An adjustable oscillator can easily change the operating frequency of the converter when the load condition changes. In the proposed converter, the integrated oscillator provides sixteen frequencies ranging from 0.1 to 200 MHz, which are selected by the main controller using 4-bit FC. For our voltage converter, the accuracy of the frequency is not critical to maintain the target output voltage, which is proven by the measurement results. Therefore, a simple low-power oscillator suffices our needs.

**Figure 3:**
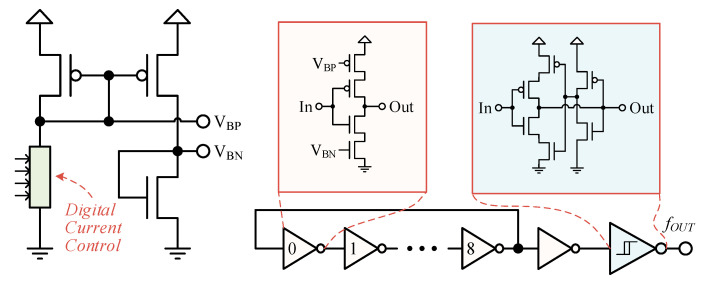
Ring oscillator with current starved inverters and biasing circuit.

Figure 3 illustrates the proposed oscillator, which is a ring oscillator consisting of nine current-starved inverters. The current-starved inverters are controlled by a biasing circuit using 4-bit digital control to tune the delay of each inverter. The digital current control has multiple current multiplication branches, one of which is selected based on the FC to provide appropriate current to the biasing circuit. A low-power Schmitt trigger is used at the output to reduce the power consumption of the subsequent buffers. 

### 2.4. Non-Overlapping Signal Driver (NOSD)

**Figure 4:**
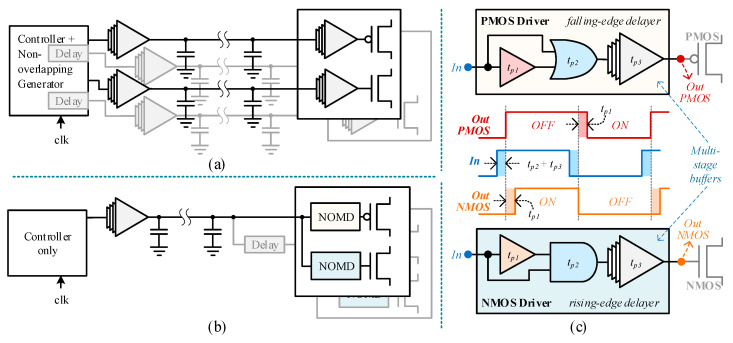
(**a**) Conventional non-overlapping architecture; (**b**) proposed non-overlapping architecture with reduced buffers and wires; (**c**) proposed non-overlapping MOSFET drivers with edge-delaying.

Conventional multiphase converters employ a shared delay controller to generate non-overlapping signals. Such architectures, however, require excessive number of long routing wires as shown in Figure 4a. In contrast, this paper proposes a distributed architecture that generates non-overlapping signals using edge delay circuits at each MOSFET switch, which is illustrated in Figure 4b. The proposed architecture can significantly reduce the number of long routing wires and their associated buffers, consequently reducing the power consumption. Each switch (NMOS and PMOS), in the capacitor banks of the proposed converter, includes a driver that generates non-overlapping control signals by delaying the turn-on transition of the switch, as shown in Figure 4c. The NMOS and PMOS drivers in the NOSDs, respectively, delay the rising and falling edges of the resulting non-overlapping signals. These drivers ensure that no short-circuit path occurs during switching, thus reducing the switching losses. From Figure 4c, it can be observed that the turn-on delay of a switch is approximately equal to the propagation delay of first buffer (*t*_*p*1_) in the drivers. This distributed topology reduces the number of wires connecting the controller with the switches. 

Consider the propagation delays of the gates in drivers as: *t_p_*_1_ for the buffer, *t_p_*_2_ for OR/AND gates, and *t_p_*_3_ for the final multi-stage buffer. The rising- and falling-edge delays of PMOS driver can be represented as:

(1) $$t_{pp-r}=t_{p2}+t_{p3},$$

(2) $$t_{pp-f}=t_{p1}+t_{p2}+t_{p3},$$

Similarly, the propagation delays for rising and falling edges through NMOS driver can be represented as:

(3) $$t_{pn-r}=t_{p1}+t_{p2}+t_{p3},$$

(4) $$t_{pn-f}=t_{p2}+t_{p3},$$

By using the above equations, we can calculate the non-overlapping periods. Upon rising-edge, NMOS turns-on and PMOS turns-off, the non-overlapping period can be formulated as:

(5) $$t_{nol-r}=t_{pn-r}-t_{pp-r},$$

(6) $$t_{nol-r}=t_{p1}+t_{p2}+t_{p3}-\left(t_{p2}+t_{p3}\right),$$

(7) $$t_{nol-r}=t_{p1},$$

Upon falling-edge, PMOS turns-on and NMOS turns-off, the non-overlapping period can be formulated as:

(8) $$t_{nol-f}=t_{pp-f}-t_{pn-f},$$

(9) $$t_{nol-f}=t_{p1}+t_{p2}+t_{p3}-\left(t_{p2}+t_{p3}\right),$$

(10) $$t_{nol-f}=t_{p1},$$

It is important that OR- and AND-gate have equal propagation delays to ensure symmetric non-overlapping periods for both the edges.

## 3. Implementation of Test Chip

**Figure 5:**
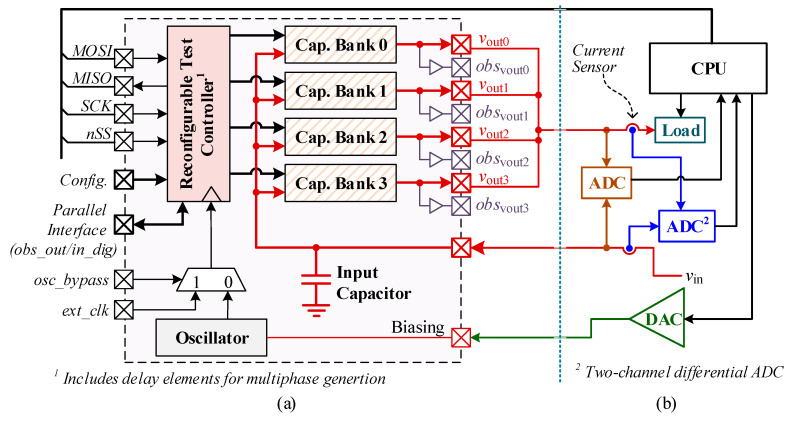
(**a**) Test chip implementation block diagram; (**b**) off-chip components for testing.

To verify the proposed architecture, we have implemented a test chip consisting of four capacitor banks, a reconfigurable test controller, and clock oscillator using 130 nm 1.5 V CMOS process, as shown in Figure 5a. The individual outputs of the four capacitor banks have been brought out of chip for measurement purposes, and they are combined on the PCB. In addition, to ensure accurate output voltage observations, four buffered outputs have been added. For test chip implementation, the oscillator does not include a digital current control circuit. Therefore, an external current Digital-to-Analog Converter (DAC) is used to drive the biasing circuit of the oscillator, as shown in Figure 5b. In addition, some components of the main controller (of Figure 2), such as OSI and VCR tables, have been moved to the off-chip CPU module to have flexibility in testing. 

### 3.1. Reconfigurable Test Controller Implementation

**Figure 6:**
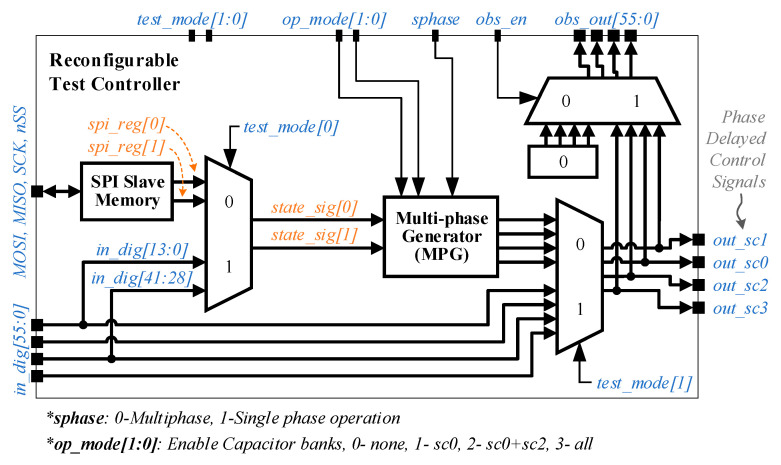
Reconfigurable test controller with interfaces, for a multiphase SC converter.

A test controller is designed with a host interface that emulates the interface to power management software. It operates the four capacitor banks at different phases (or in phase) and can be programmed through its host interface, as shown by the simplified block diagram in Figure 6. In addition to its main functions, we implemented various test and fallback modes in the controller for test purposes. The next entry of the OSI table provides the state information for each capacitor bank, which is stored in VCR state registers (state_reg[0] and state_reg[1]). The VCR state registers’ values are used to generate control signals for each attached capacitor bank.

The multi-phase generator (MPG) block reads the state signals and operates the capacitor banks by supplying phase-delayed signals. The four operation modes of MPG are listed in Figure 6, which allows one, two or all four converters to operate concurrently. It is also possible to operate two or four capacitor banks in phase, by asserting *sphase* input, to compare the performance of single-phase and multi-phase operations.

The test controller has been synthesized using Synopsys design tools targeting 250 MHz. The final implemented layout of the controller has an active area of 590 μm × 15 μm. The aspect ratio of the test controller was chosen to minimize the routing overhead when connecting the controller to a stack of four capacitor banks.

### 3.2. Capacitor Banks

**Figure 7:**
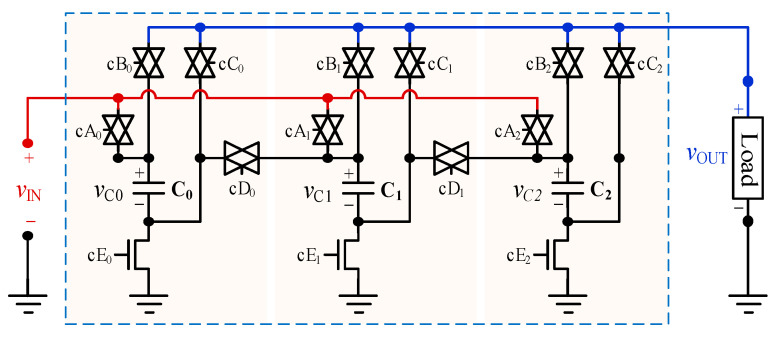
Capacitor bank circuit diagram with three switched capacitor branches.

For the test chip, we implemented four identical series-parallel switched capacitor banks. A circuit block diagram of one bank is shown in Figure 7. Each bank has three flying capacitors which provide five buck voltage conversion ratios (1/4, 1/3, 1/2, 2/3, 3/4). For our target load current range, we chose 80 pF for each flying capacitor and 160 pF for the load decoupling capacitor in each capacitor bank.

**Figure 8:**
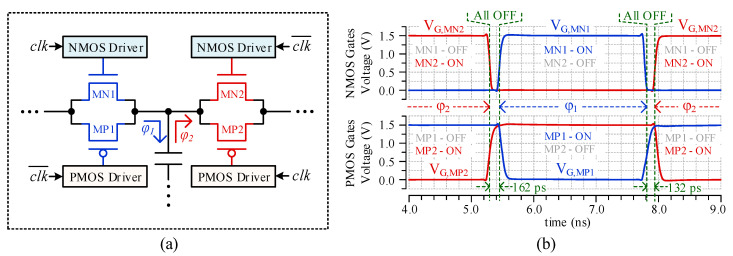
(**a**) Transmission gate switches controlled by Non-Overlapping Signal Drivers (NOSDs). For example, the blue transmission gate indicates cA_0_ and the red transmission gate indicates cB_0_ of Figure 7. (**b**) Post-layout simulation of the NOSDs, demonstrating non-overlapping control to mitigate short-circuit losses.

Figure 8 illustrates an example of non-overlapped switching operation. Figure 8a shows example MOSFET switches corresponding to transmission gates cA0 and cB0 of Figure 7. The NMOS and PMOS drivers are part of the NOSD of a capacitor bank shown in Figure 4c. Figure 8b shows the post-layout simulation result of the circuit in Figure 8a. It reveals that the NMOS and PMOS gates have a non-overlapped period when transitioning between ϕ_1_ and ϕ_2_, which ensures that no short-circuit path occurs while switching. For the test chip, the non-overlapped period introduced by the NOSD varies from 130 to 200 ps, depending on the potential on the source and drain terminals of the MOSFET being driven.

### 3.3. Overall Test Chip Implementation

**Figure 9:**
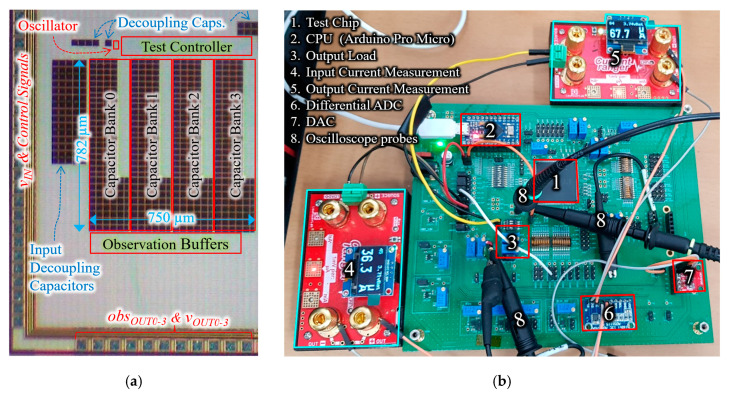
(**a**) Die microphotograph of the implemented proposed controller with test controller and oscillator; (**b**) measurement setup showing test PCB and off-PCB current measurement devices.

Complete layout (including pads) of the overall test chip is shown in Figure 9a, where the converter occupies an area of 0.59 mm^2^. The voltage supplies are located on the top left, whereas analog/power outputs (v_out_ and obs_vout_) are located on the bottom. Decoupling capacitors are added to the voltage supplies of MOSFET drivers, oscillator and reconfigurable test controller, as shown in Figure 9a. The test chip shown in Figure 9a occupies total area of 1.76 mm^2^ (1.56 mm × 1.13 mm), including test circuits and pads. For this implementation, only metal–insulator–metal (MIM) capacitors are used for capacitor banks to minimize switching losses [12] caused by the bottom plate capacitance (C_bot.,mim_ = 0.0025 × C). To reduce the overall area, a metal–oxide–semiconductor (MOS)+MIM stack can be safely used as load capacitors, without sacrificing conversion efficiency. Using an MOS+MIM stack as a load capacitor, the area can be reduced by 21% (from 0.59 mm^2^ to 0.461 mm^2^), compared to an MIM-only load capacitor. Moreover, our analysis also shows that replacing all capacitors by an MOS+MIM stack yields a 54% reduction in area (from 0.59 mm^2^ to 0.266 mm^2^) at the expense of conversion efficiency.

## 4. Results

The measurement setup to verify the implemented test-chip is shown in Figure 9b. For accurate current measurements at the input and output of the converter, two current sensors [13] and high-resolution Analog-to-Digital Converters (ADCs) are employed. To adjust the frequency of the on-chip oscillator, a 12-bit DAC is used. For the sake of testing, we have split the load and the power management CPU of Figure 2 into separate external components. Two series variable resistors are acting as a load, one of which can be bypassed to toggle between heavy and light load conditions. A CPU module is used to run a power management program and governs the on-chip controller, the external DAC, and the load. The CPU module also reads the current measurements using the external ADC and the voltage measurement using the internal ADC.

**Figure 10:**
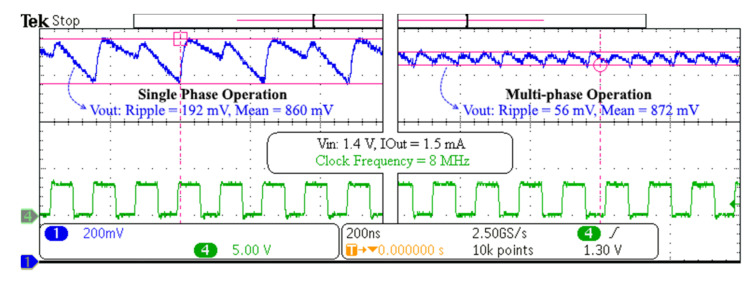
Single-phase vs. multi-phase operation of the implemented converter.

The first measurement result is the difference between single-phase and multi-phase operations, which is shown in Figure 10. It can be observed that the ripple voltage of the multi-phase case is significantly reduced compared with the single-phase. Moreover, the average output voltage of the multi-phase case has increased, resulting in improved efficiency.

**Figure 11:**
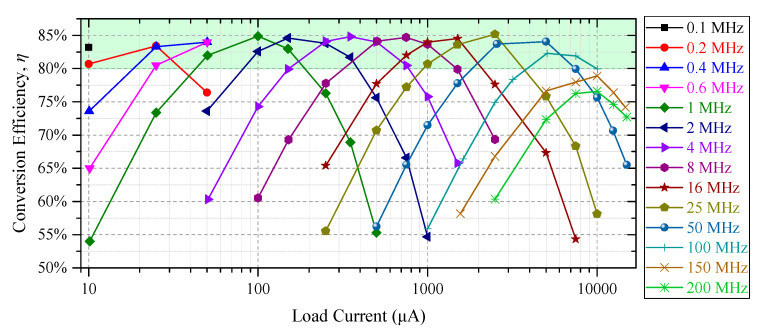
Measurement results demonstrating wide load range performance over various switching frequencies for a voltage conversion ratio of 3/4.

To evaluate the performance of the implemented converter over a wide load range, a set of measurement are performed by reconfiguring the load and the oscillator frequency. The load is configured by adjusting the off-chip resistor, while the converter takes 1.4 V as input. The oscillator frequency is adjusted by the power management CPU via the DAC. The results provided in Figure 11 demonstrate that the converter is able to maintain a high efficiency of above 80% for the targeted load range from 10 μA to 10 mA, by adjusting the switching frequency using the on-chip oscillator. Furthermore, the converter provides a conversion efficiency of 74% at 15 mA, while operating at 150 MHz. In the test chip, where the source and load are off-chip, the conversion efficiency drops above 7.5 mA due to a large voltage drop across the bonding and metal wires. However, for true on-chip implementation, this efficiency will be maintained above 7.5 mA as well. At high frequencies, the power losses (switching) increase while the power delivered slowly saturates due to conduction losses. This results in the degradation of the conversion efficiency at higher frequencies.

**Figure 12:**
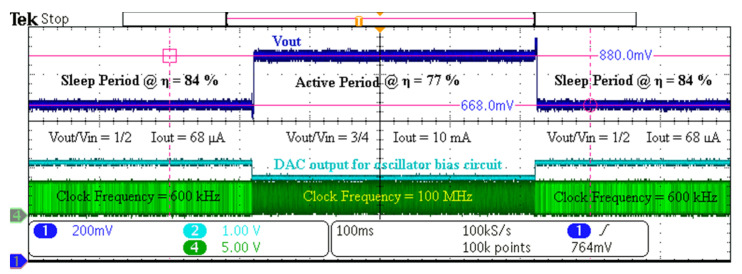
Measurement result for transitions between sleep and the active period of the load.

To verify the transition operation between heavy (active) and light (sleep) load, the CPU module momentarily changes the load and the frequency. Figure 12 shows the transition operation when the load switches between 68 µA and 10 mA by switching the frequencies between 600 kHz and 100 MHz, respectively. Usually in an active state, a higher voltage is needed to support fast computation in the load (CPUs or SoCs) than the sleep state [14,15]. Therefore, in the test for Figure 12, we configured the voltage conversion ratio to 1/2 for sleep and 3/4 for active period.

**Figure 13:**
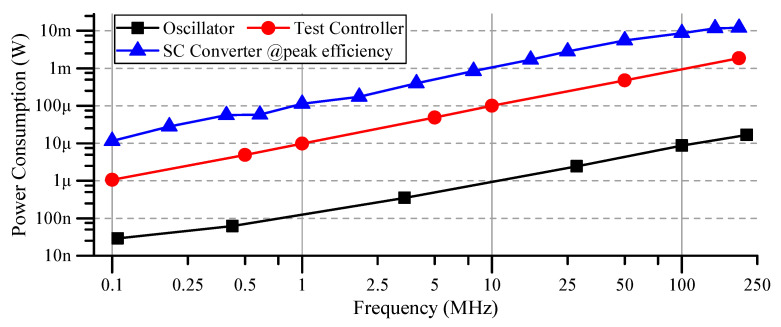
Test-chip power consumption at various operating frequencies.

Figure 13 reflects the power consumption of the main blocks of the test chip, over the supported frequency range. It can be observed that the power consumption of the oscillator and the controller decreases with the decrease in frequency, allowing for higher conversion efficiencies at low currents. The power consumption of the SC converter does not include the power supplied to the test controller. Since the test controller is designed to provide many additional test features, its power consumption does not truly reflect an optimized main controller. Moreover, the on-chip analog buffers (used for output voltage observation) consume around 20 mW.

## 5. Discussion

The proposed SC converter’s advantage—the ability to transition smoothly between light and heavy loads, while maintaining high conversion efficiency—makes the converter well suited to loads that frequently change operating states. We have proposed a figure of merit (FOM) to evaluate a converter for wide load range operation, using:

(11) $$\mathrm{FOM}=\left(\eta_{Max}+\eta_{I,Max}+\eta_{I,Min}\right)\times \log\left(\frac{I_{Max}}{I_{Min}}\right),$$

Here, $\eta_{Max}$ is the peak conversion efficiency, while $\eta_{I,Max}$ and $\eta_{I,Min}$ are the conversion efficiencies at maximum and minimum load, respectively. Table 1 compares the design parameters and performance of our work with previously reported solutions. Table 1 shows that the proposed converter maintains higher efficiency compared to other works [6,10,16]. This is accomplished by the ability of the proposed converter to operate at a much wider switching frequency. Due to the limitation of existing feedback-based PFM, only limited f_max_/f_min_ of 132 is used in [6], while the proposed converter allows for an extremely large f_max_/f_min_ up to 1000. The capacitor banks used in this implementation offer five buck voltage conversion ratios, higher than the works in [5,6,9,10,11,16]. To further reduce the output ripple while having the wide load range, our architecture allows the number of phases to be increased to match [5,6]. The proposed FOM reveals the superior performance of 7.44, compared to existing works. Though the converter proposed in [9] reaches an FOM of 6.74, it uses off-chip flying capacitors and two discrete converters for sleep and active operation.

While this paper is focused on the proactive PFM, it is not limited only to proactive PFM with prior schedules. We can combine the proactive PFM with existing feedback-based PFM methods [6,11] to take advantage of both methods. Then, the feedback can allow finer adjustments in frequency, whereas the proactive PFM provides a wider frequency range, which is expected to provide further improvement.

## 6. Conclusions

This paper has proposed and evaluated a wide load range SC converter aiming on sensor nodes, where the load power requirements varies based on a schedule. The proposed voltage converter is proactively reconfigured to ensure high conversion efficiency, for various load current and voltage requirements. The converter utilizes multiphase operation to mitigate output ripple voltage and phase frequency modulation using an integrated oscillator to maintain high conversion efficiency across the load range. In addition, the converter employs the proposed distributed non-overlapping generation to reduce area and power consumption. The measurements demonstrate above 80% efficiency for output current ranging from 10 μA to 10 mA. Due to its wide load range, the converter is a strong candidate for driving sensor nodes, where the power requirements often change by orders of magnitude between sleep and active state. The converter architecture allows for upscaling the number of phases/capacitor banks to further reduce output ripple. Moreover, the converter architecture can be evolved to incorporate existing feedback-based PFM to fine-tune its oscillator frequency against minor load variations.

## Figures and Tables

**Table 1:** Comparison of performance with state of the art.

Characteristics	[6]	[9]	[10]	[16]	This Work
Tech (nm)	65	800	45	130	130
No. of VCRs	3	1	1	1	5
Vin (V)	1.6–2.2	3.6–4.2	1.8	3.3	1.5
Vout (V)	0.6–1.2	1.7–2.1	0.8–1	1.2–1.5	0.4–1.1
$I_{L,Min}$ (mA)	5 ^b^	0.005	0.1	1 ^b^	0.01
$I_{L,Max}$ (mA)	125 ^b^	2	10	53	10
$C_{fly}$ (pF)	4797	440,000 ^a^	534	2176	960
$C_{L}$ (pF)	2000	1,000,000 ^a^	700	1000	640
Ripple (mV)	2.2–30	100	<50	8–55	56
$\eta_{Max}$ (%)	80	94	69	73	85
$\eta_{I,Max}$ (%)	73 ^b^	85	48	73 ^b^	80
$\eta_{I,Min}$ (%)	71 ^b^	80	65	44 ^b^	83
FOM	3.13	6.74	3.64	3.28	7.44
Area (mm^2^)	0.842	N/A	0.16	2.04	0.59
Freq. (MHz)	0.25–33	0.05	30	40	0.1–200

^a^ Off-chip Component. ^b^ Approximated values from graphs.
